# Temperature Field Measurement of Photovoltaic Module Based on Fiber Bragg Grating Sensor Array

**DOI:** 10.3390/ma15155324

**Published:** 2022-08-02

**Authors:** Guoli Li, Fei Feng, Fang Wang, Bo Wei

**Affiliations:** 1 School of Mechanical and Electrical Engineering, Jinling Institute of Technology, Nanjing 211169, China; fengfei4515@126.com (F.F.); wangfang@jit.edu.cn (F.W.); 2 Wuxi Brillouin Electronic Technology Co., Ltd., Wuxi 214131, China; wb@buliyuan.com

**Keywords:** fiber optics, photovoltaic module, fiber Bragg grating sensor array, temperature field, wavelength division multiplexing, space division multiplexing

## Abstract

Studying the temperature field of photovoltaic modules is important for improving their power generation efficiency. To solve the problem of traditional sensors being unsuitable for measuring the spatial temperature field, we designed a real-time detection scheme of the photovoltaic module temperature field based on a fiber Bragg grating (FBG) sensor array. In this scheme, wavelength division multiplexing and space division multiplexing technologies were applied. The multi-channel FBG sensor strings were arranged on the surface and in the near field of the photovoltaic module. Different FBG strings were selected through optical switches, and the wavelength of the FBG string was addressed and demodulated using the tunable laser method and a peak-seeking algorithm. A measurement experiment of the photovoltaic module temperature field was carried out in an outdoor environment. The experimental results showed that the fluctuation law of the photovoltaic module surface and near-field temperature is basically consistent with that of solar radiation power. The temperature of the photovoltaic module decayed from the surface to space. Within 6 mm of the photovoltaic module surface, the temperature sharply dropped, and then the downward trend became flat. The lower the solar radiation power and the higher the wind speed, the faster the temperature decay. This method provides technical support for measuring the temperature field of a photovoltaic module and other heat source equipment.

## 1. Introduction

Solar energy is one of the most used clean energy sources. In recent years, the photovoltaic industry has rapidly developed under the pressure of global carbon reductions and the fossil fuel energy crisis. A photovoltaic cell is a component that converts light energy into electric energy. The photoelectric conversion efficiency of a photovoltaic cell is the main factor that determines the power generation capacity of a photovoltaic system. Taking the crystalline silicon photovoltaic cell as an example, its photoelectric conversion efficiency limit is about 30%, but the actual conversion efficiency is 10%~26% [1]. Photovoltaic cells convert a small part of the absorbed incident solar energy into electric energy. However, most of the remaining energy is in the form of thermal energy, which causes the temperature to rise. The rise in photovoltaic cell temperature creates potential safety hazards such as photovoltaic cell failure caused by the hot spot effect [2,3]. Additionally, photoelectric conversion efficiency decreases with the increase in working temperature [4,5].

An effective cooling method can reduce the temperature of a photovoltaic module and improve its photoelectric conversion efficiency. To improve the cooling mode and power generation efficiency of a photovoltaic module, studying the spatial temperature field distribution and its influencing factors is important.

At present, the research on temperature field detection has mainly focused on the surface temperature of the photovoltaic module. Three main methods are commonly used to detect the temperature of a photovoltaic module:

(1) The contact measurement method based on thermal resistance, thermocouples, and other electrical sensors: To avoid the shadow of the sensor, the electrical sensor is usually placed on the surface of the photovoltaic cell backplane for temperature measurement. Bohorquez et al. [6] used a DS18B20 digital temperature sensor to measure the temperature of photovoltaic facilities. The sensor was calibrated and compared with a Pt100 thermal resistance sensor. The deviation between the developed system and the system based on the standard Pt100 was less than ±0.4 °C. Martínez et al. [7] used a single bus digital temperature sensor to measure the temperature of photovoltaic facilities.
(2) The noncontact measurement method based on visible light and infrared imaging: Tsanakas et al. [8] collected the infrared thermal image of a photovoltaic array for image processing, and selected the Canny edge detection operator to identify the hot spot effect module. Bu et al. [9] established an experimental system of pulse electric infrared thermal imaging (PEIT). The results showed that the PEIT algorithm could effectively detect the defects of photovoltaic cells. For obtaining a large field of view of the photovoltaic array image, Mao et al. [10] proposed an automatic splicing algorithm for infrared photovoltaic images based on a fast robust feature detection operator. It performs the full-automatic splicing process from image sequence to panorama. Niazi et al. [11] used the texture and gradient histogram features of photovoltaic module thermal images for classification; the machine learning algorithm was trained to detect hot spots on photovoltaic panels.
(3) The measurement method based on the electrical characteristics of a photovoltaic module. Kim et al. [12] proposed an active hot spot detection method. The results showed that the hot spot in a single cell can increase the capacitance and DC impedance. Ma et al. [13] proposed a hot spot fault diagnosis method based on the photovoltaic module I–V curve. Ghanbari [14] detected the shading hot spot effect by calculating the equivalent DC impedance (EDCI) of a photovoltaic module. Wang et al. [15] proposed an improved fast R-CNN infrared hot spot image detection method. It improved the recognition accuracy of hot spots. Jia [16] proposed a multisensor fault detection and location method based on an improved BP neural network.

For large-area photovoltaic modules, the above methods need a large number of sensors to be arranged or a large number of images to be collected, which is high-cost and involves complex data processing, and results in low measurement efficiency and poor real-time performance. The third method is usually used to monitor abnormal temperatures of photovoltaic modules; the output voltage, current, and other parameters of photovoltaic modules must be collected in real time by building auxiliary circuits. These data are analyzed by using mathematical statistical models.

However, the above methods are not suitable for the measurement of spatial temperature fields. For measuring the spatial temperature field of a photovoltaic module, the selected sensor must be of a sufficiently small size.

The FBG temperature sensor is a new type of sensor. Compared with traditional sensors, it has many advantages, such as small mass and volume, corrosion resistance, antielectromagnetic interference, easy multiplexing, easy remote operation, and so on. FBGs with different central wavelengths can be engraved at different positions of an optical fiber. By cascading multiple FBG strings, a sensing array is formed. This is especially suitable for distributed measurement. In recent years, researchers have carried out extensive research on temperature measurement with FBG sensor arrays [17,18,19,20,21,22].

In a recent study, we analyzed the surface temperature distribution of a photovoltaic module by a FBG sensor, and realized the detection of hot spots [23]. On the basis of previous work, the FBG sensor array was used to measure the multipoint temperature of near space of a photovoltaic module. The distribution and variation laws of the spatial temperature field of a photovoltaic module were studied. The relationship with factors such as solar radiation power and wind speed was discussed. The results showed that the proposed method has improved temperature measurement efficiency for photovoltaic modules, as well as provides a reference for the measurement of the spatial temperature field in other fields.

## 2. Photovoltaic Module Temperature Field Analysis

### 2.1. Energy Input and Output of a Photovoltaic Module

**Figure 1:**
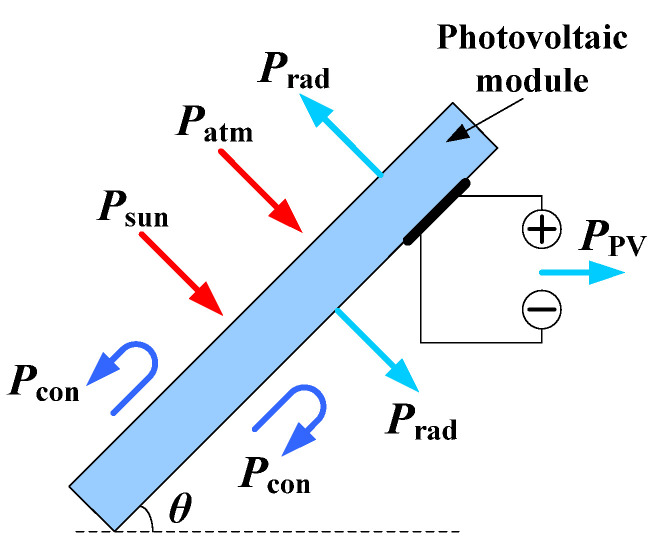
Schematic diagram of energy input and output of photovoltaic module.

The photovoltaic module can absorb energy from incident sunlight and the environment, and release energy through the photovoltaic effect and heat exchange process. The photovoltaic module is shown in Figure 1. The power equation is shown in Equation (1):

(1) $$P_{\mathrm{sun}}+P_{\mathrm{atm}}=P_{\mathrm{rad}}+P_{\mathrm{con}}+P_{\mathrm{PV}}$$

where $P_{\mathrm{sun}}$ is the power absorbed by a photovoltaic module from solar radiation, $P_{\mathrm{atm}}$ is the power absorbed by a photovoltaic module from environmental radiation, $P_{\mathrm{rad}}$ is the radiant thermal power of a photovoltaic module, $P_{\mathrm{con}}$ is the thermal dissipation power of the photovoltaic module by nonradiative means (mainly thermal convection), and $P_{\mathrm{PV}}$ is the electrical output power of a photovoltaic module.

When the solar radiation power fluctuates, the temperature of the photovoltaic module accordingly changes as the parameters change on both sides of Equation (1). At the same time, the power released into the air changes, causing near-field temperature changes in the module. In addition, the distribution of the photovoltaic module near-field temperature is affected by the ambient wind speed. Therefore, the temperature field is affected by solar radiation power, ambient temperature, wind speed, photovoltaic cell performance, and other factors.

### 2.2. Photovoltaic Module Temperature Model

Ross et al. [24] conducted experimental research on the nominal temperature of solar cells. They = proposed a mathematical model to predict the surface temperature of a photovoltaic module:

(2) $$T_{\mathrm{cell}}=T_{\mathrm{air}}+\frac{T_{\mathrm{NOC}}-20}{800}\cdot S$$

in which $T_{\mathrm{cell}}$ is the photovoltaic module temperature, °C; $T_{\mathrm{air}}$ is the ambient temperature, °C; $T_{\mathrm{NOC}}$ is the nominal temperature of the photovoltaic module, °C, which refers to the temperature of the photovoltaic module under the conditions of an ambient temperature of 20 °C, solar radiation power of 800 W/m^2^, and wind speed of 1 m/s; S is the solar radiation power of the photovoltaic module, W/m^2^.

Based on the above nominal model, many scholars have carried out many studies. Many photovoltaic module temperature prediction models such as those by Skoplaki et al. [25], Mattei et al. [26], Sandia et al. [27], Faiman [28], and Muzathik et al. [29,30] have been proposed. The multiple linear regression equation model proposed by Muzathik is shown in Equation (3):

(3) $$T_{\mathrm{cell}}=aT_{\mathrm{air}}+bS-cv_{\mathrm{wind}}+d$$

in which a, b, c, and d are the system specific linear regression coefficients; $v_{\mathrm{wind}}$ is the wind speed.

The hot spot effect can produce abnormal temperatures in a photovoltaic module. A photovoltaic module is made of photovoltaic cells in series, parallel, and packaged. When a photovoltaic cell is damaged or blocked by dust, leaves, etc., its photogenerated current decreases and becomes a hot spot cell. As all cells in the photovoltaic module work at the same current intensity, the hot spot cell is reversely biased. As a result of the large power dissipation from the shaded cell, extremely high heat is generated and accumulated resulting in a sharp temperature rise, causing the hot spot effect.

### 2.3. Analysis of Spatial Temperature Field of Photovoltaic Module

Thermal radiation and thermal convection have a strong influence on the spatial temperature field near the surface of a photovoltaic module. The thermal power released from a photovoltaic module surface through thermal radiation and thermal convection is:

(4) $$P=P_{\mathrm{rad}}+P_{\mathrm{con}}$$

where $P_{\mathrm{rad}}$ is the thermal power released by thermal radiation, W; $P_{\mathrm{con}}$ is the thermal power released by thermal convection, W.

The thermal power released by thermal radiation is:

(5) $$P_{\mathrm{rad}}=h_{r}(T_{\mathrm{cell}}^{}-T_{\mathrm{air}}^{})A$$

where A is the heat radiation surface area, m^2^; $h_{r}$ is the equivalent radiative heat transfer coefficient, W/(m^2^·K); its value is calculated by the following equation:

(6) $$h_{r}=\varepsilon \sigma (T_{\mathrm{cell}}^{}+T_{\mathrm{air}}^{})(T_{\mathrm{cell}}^{2}+T_{\mathrm{air}}^{2})$$

in which ε is the surface emissivity of a photovoltaic module, σ is the Stefan Boltzmann constant, with a value of 5.67 × 10^−8^ W/(m^2^·K^4^).

The thermal power released by thermal convection is:

(7) $$P_{\mathrm{con}}=h_{c}(T_{\mathrm{cell}}-T_{\mathrm{air}})A$$

in which $h_{c}$ is the convective heat transfer coefficient, W/(m^2^·K).

In engineering applications, the convective heat transfer coefficient can generally be calculated by the formula shown in the following equation [31]:

(8) $$\begin{array}{c} R_{a}=G_{r}P_{r}=\frac{g\beta (T_{\mathrm{cell}}-T_{\mathrm{air}})L^{3}}{\mu k}\\ N_{u}=aR_{a}^{b}\\ h_{c}=\frac{N_{u}k}{L} \end{array}\}$$

where $R_{a}$ is the Rayleigh number, $G_{r}$ is the Grashof number, $P_{r}$ is the Prandtl number of air, μ is the kinematic viscosity coefficient of air, k is the thermal conductivity of air, g is the acceleration of gravity, β is the coefficient of thermal expansion of air, L is the longitudinal length of the photovoltaic module placed at an inclination, and $N_{u}$ is the Nusselt number.

For the convective heat transfer at a free boundary, the surface heat transfer coefficient of a photovoltaic module can be calculated by the following empirical equation [32]:

(9) $$h_{c}=5.7+3.8v_{w}$$

where $v_{w}$ is the ambient wind speed, m/s.

According to Equations (5) and (7), the following equation can be obtained:

(10) $$P=P_{\mathrm{rad}}+P_{\mathrm{con}}=(h_{c}+h_{r})(T_{\mathrm{cell}}^{}-T_{\mathrm{air}}^{})A=h(T_{\mathrm{cell}}^{}-T_{\mathrm{air}}^{})A$$

where $h=h_{c}+h_{r}$ is the surface thermal power release coefficient of the photovoltaic module.

In pure natural convection, the flow intensity caused by buoyancy can be expressed by the Rayleigh number $R_{a}$. In outdoor environments, when the operating temperature of the photovoltaic module placed at an angle of 30~80° is 30~100 °C, $10^{8}<R_{a}<10^{10}$ [33]. In this case, the buoyancy-driven convection is in the transition stage between laminar flow and turbulent flow, and the temperature field changes near the surface of the photovoltaic module are very complex at this stage. The temperature difference between the air and photovoltaic module surface results in a large temperature gradient near the module surface affected by laminar flow. However, the temperature change is relatively flat in the turbulent layer.

When the photovoltaic module works outdoors, the solar radiation power, wind speed, and ambient temperature have a strong impact on its temperature field. As the main factor affecting the ambient temperature is solar radiation power, in this study, we considered the influence of solar radiation power and ambient wind speed on the temperature field.

## 3. Temperature Detection Mechanism of FBG Sensor Array

### 3.1. FBG Temperature Sensing Principle

FBG is an optical fiber passive device whose refractive index is periodically modulated in the fiber core. It can reflect light of specific wavelengths, which is the FBG center wavelength $\lambda_{B}$. $\lambda_{B}$ is related to the grating period Λ and the effective refractive index $n_{\mathrm{eff}}$ of the fiber core, shown in Equation (11):

(11) $$\lambda_{B}=2n_{\mathrm{eff}}\Lambda$$

FBG is affected by the external temperature and axial stress, the photothermal effect caused by temperature changes the effective refractive index, the thermal expansion coefficient changes the grating constant, and the photoelastic effect under stress changes the refractive index. Therefore, the relative displacement of the central wavelength is:

(12) $$\frac{\Delta \lambda_{B}}{\lambda_{B}}=(\alpha +\xi )\Delta T+(1-P_{e})\varepsilon_{z}$$

in which $\alpha =\frac{1}{\Lambda }\frac{\partial \Lambda }{\partial T}$ is the thermal expansion coefficient of optical fiber, $\xi =\frac{1}{n_{\mathrm{eff}}}\frac{\partial n_{\mathrm{eff}}}{\partial T}$ is the thermal optical coefficient of optical fiber, $P_{e}=n_{\mathrm{eff}}^{2}[P_{12}-\upsilon (P_{11}+P_{12})]/2$ is the effective elastic-optic coefficient, and υ is the optical fiber Poisson’s ratio.

If FBG is prevented from being disturbed by stress, there is a linear relationship between $\Delta \lambda_{B}$ and ΔT. The temperature change can be determined by detecting the displacement of the wavelength.

### 3.2. Temperature Measurement System Based on FBG Array

**Figure 2:**
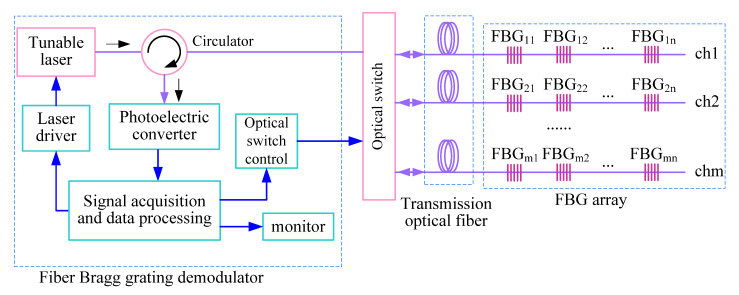
Structure diagram of temperature measurement system based on FBG sensor array.

Optical multiplexing technology is an important technical means to build a distributed FBG sensor system. The structure of the temperature measurement system is shown in Figure 2. FBGs are connected in series by wavelength division multiplexing on each channel. The number of FBGs in series depends on the scanning wavelength range of the demodulator and the operating wavelength range of each FBG. The space division multiplexing method is used between different sensing channels to expand the capacity of FBG networks [34].

The wavelength of a narrow-band light source output can be varied within a certain range by using a tunable laser. The laser scanning step size and frequency can be controlled by a driver. A narrow-band laser is emitted to an FBG array through a circulator; when the wavelength of the light source is consistent with the central wavelength of an FBG, the light intensity of the reflected signal is the largest. When the reflected light signal reaches the photoelectric converter through the circulator, it is converted into an electrical signal. A data processing computer collects electrical signals. The peak value of the signal voltage is obtained by the peak-seeking algorithm; it performs wavelength demodulation and positioning of the FBG. The measured temperature is calculated by comparing the variation in the central wavelength of each FBG sensor. The optical switch can select different sensing channels. The FBG array is arranged on the surface of the photovoltaic module and in nearby space to measure the temperature. The system realizes the identification and positioning of each sensor of the FBG array through wavelength division multiplexing and space division multiplexing technologies.

## 4. Experimental Equipment and FBG Calibration

**Figure 3:**
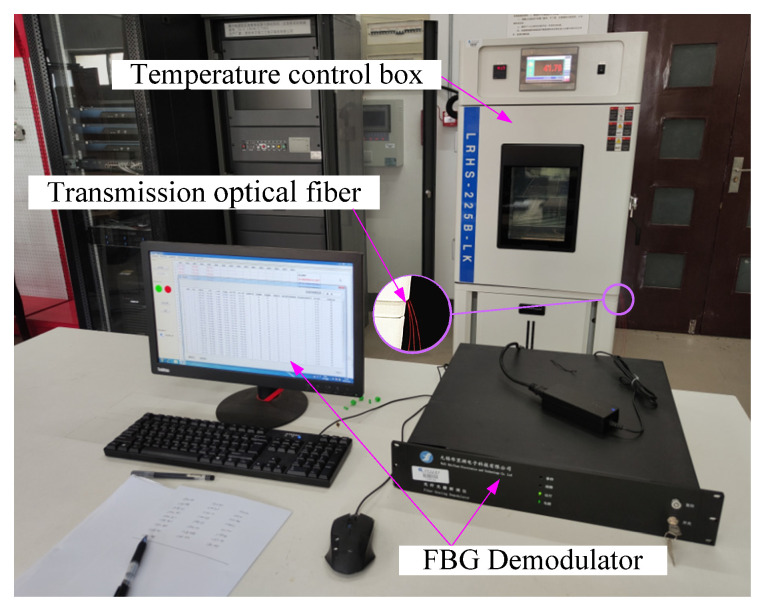
Fiber Bragg grating calibration experiment.

The FBG demodulation equipment used in the experiment was a BLY-FBG-5S demodulator (Wuxi Brillouin Electronic Technology Co., Ltd., Wuxi, China), as shown in Figure 3. The light source band of the demodulator was 1525∼1565 nm; the wavelength resolution was 0.1 pm. The scanning frequency was 100 Hz, and there were 8 sensing channels.

In the experiment, a thermal infrared camera was used to measure the surface temperature of the photovoltaic module. The measurement results were compared with the temperature measured by the FBG sensors. The infrared camera used was a VarioCAM^®^HD inspect 980 infrared thermal imager (InfraTec company, Dresden, Germany).

The size parameter of the photovoltaic module was 960 × 480 mm, and the power of the photovoltaic power generation system was 800 W. Three FBG sensor strings were used in the experiment. Each string was engraved with 12 gratings, for a total of 36 FBGs. The length of the FBG grating area was 10 mm and the minimum bandwidth of the FBG was 2 nm.

The number format of each FBG sensor was FBGmn. The string number varied in the range of 1~3, and n was defined as the serial number of FBG in the string with a range of 1~12.

**Figure 4:**
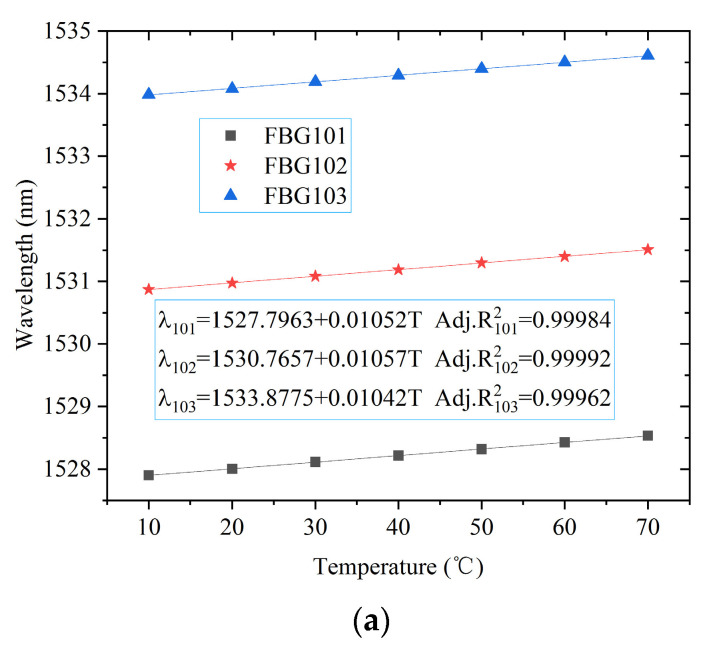

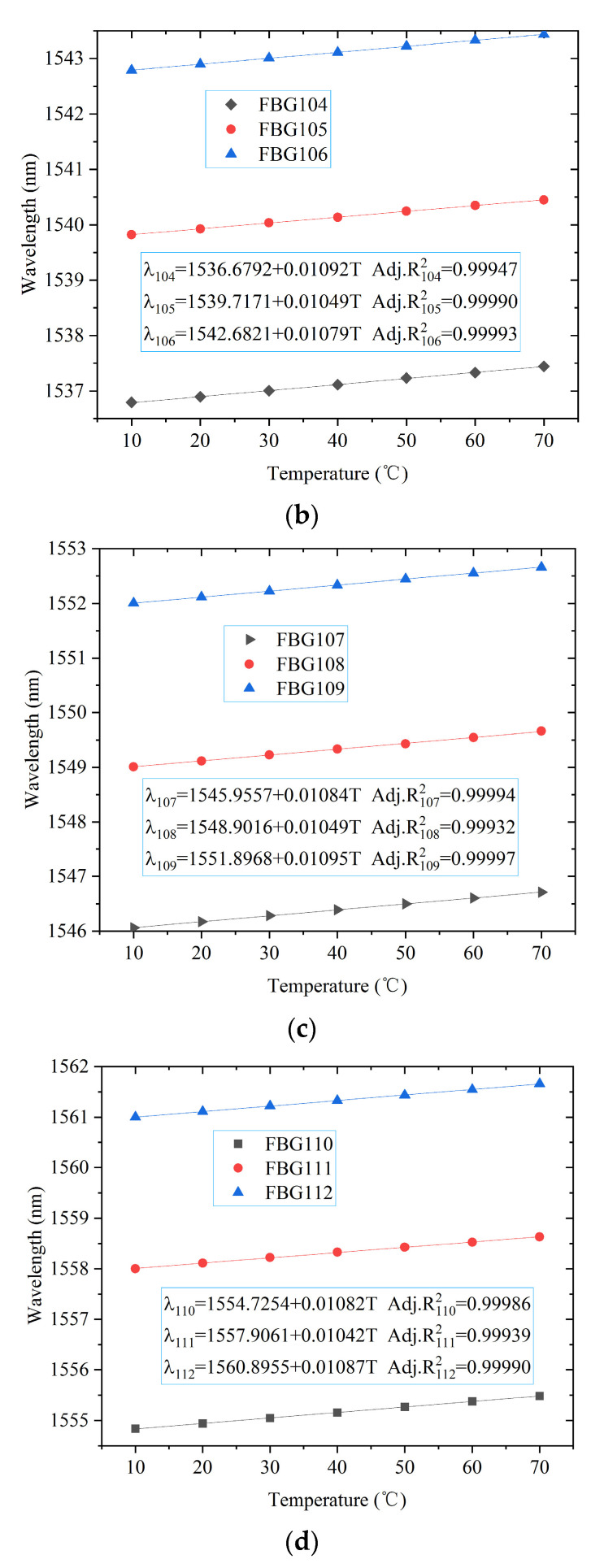
Calibration curves of FBGs: (**a**) FBG101-FBG103; (**b**) FBG104-FBG106; (**c**) FBG107-FBG109; (**d**) FBG110-FBG112.

Before the experiment, it was necessary to calibrate the temperature of each FBG. The FBG sensors were put into a temperature control box. The temperature of the control box was set from 10 to 70 °C. The center wavelengths of the FBGs were recorded every 10 °C, as shown in Figure 3. The calibration curve of the first FBG string is shown in Figure 4. The calibration results showed that the temperature change of each FBG was basically linear with the central wavelength displacement in the measured temperature range.

## 5. Experiment and Result Analysis

### 5.1. Surface Temperature Measurement of Photovoltaic Module

**Figure 5:**
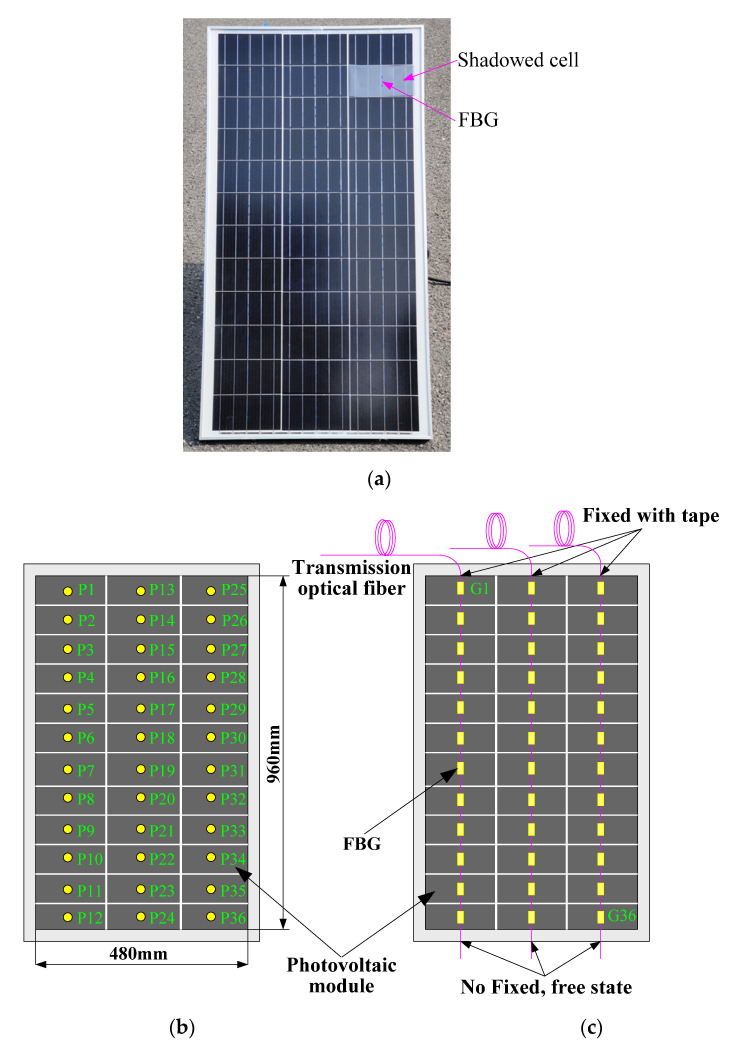
Layout of FBG sensors: (**a**) photovoltaic module; (**b**) layout of temperature measuring points; (**c**) sensors layout.

The experimental platform was built according to Figure 2, as shown in Figure 5. The FBG strings were pasted onto the photovoltaic module with thermal conductive silicone grease. FBG sensors G1 G36 were pasted to points P1~P36, respectively, and each point corresponded to a photovoltaic cell.

The FBG string was pasted on the photovoltaic module and arranged up and down in the experiment. The upper end of the fiber was fixed with adhesive tape, and the lower end was in a free state. In this way, the FBG was prevented from being affected by the stress.

**Figure 6:**
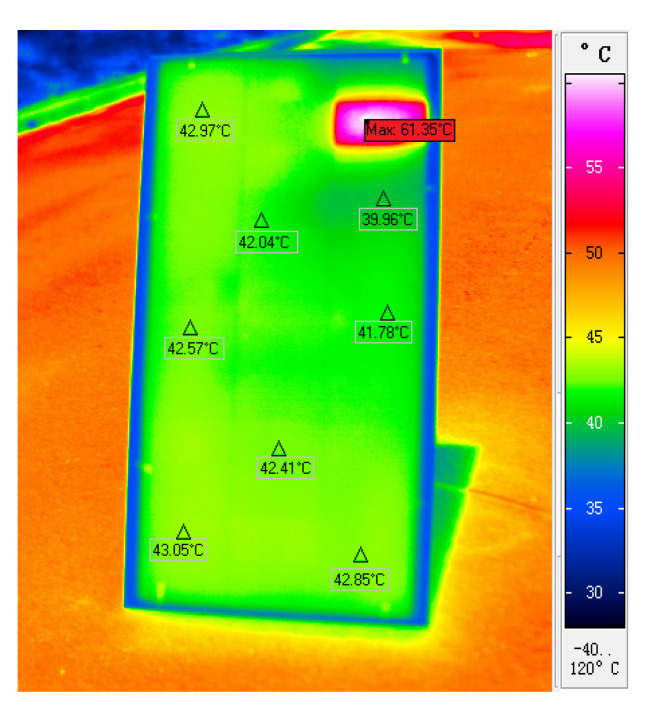
Infrared thermal image of photovoltaic module.

The surface temperature measurement experiment of the photovoltaic module was carried out in an outdoor sunny environment. The module was inclined at a 45° angle, and a plastic film with a light transmittance of 0.5% was used to cover 100% of the area of the cell at the P26 point to simulate the hot spot effect. The infrared thermal image of the photovoltaic module collected by the infrared camera is shown in Figure 6. During the experiment period, the southeasterly wind was class 2. Therefore, the temperature on the right side of the module was lower due to the influence of the wind.

**Figure 7:**
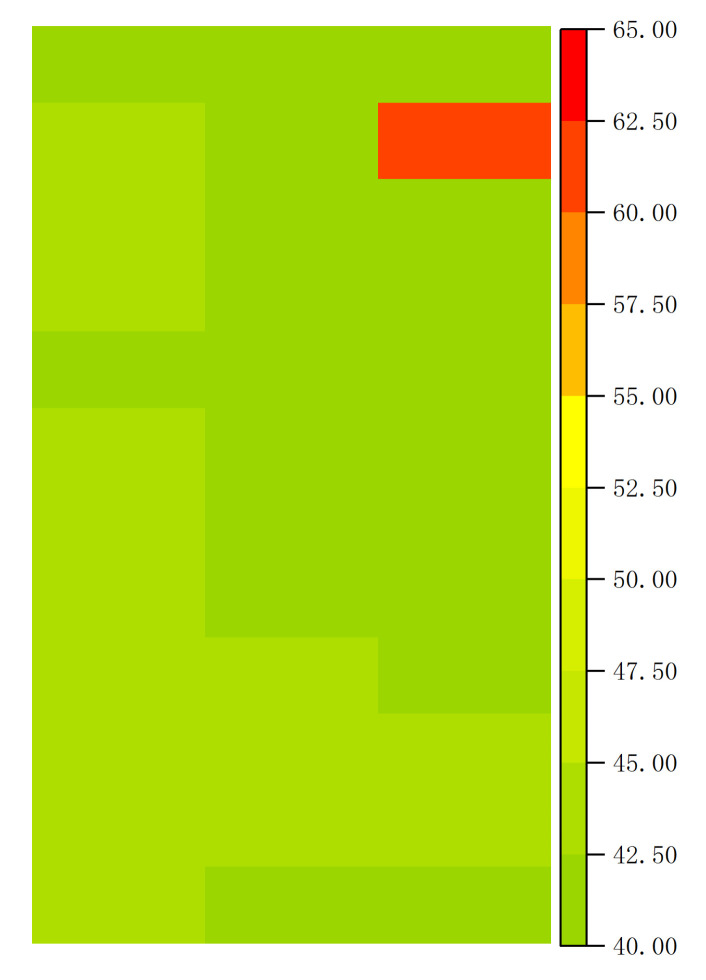
Isotherm diagram of FBG temperature measurement results.

At 9:00 a.m., the temperature data measured by the FBGs at each point were as shown in Table 1. The isotherm (color fill) drawn according to Table 1 is shown in Figure 7.

Table 1, Figure 6, and Figure 7 show that the temperature at point P26 was significantly higher than that in the surrounding area, which indicated that the cell at this position had a hot spot effect. The temperature measurements of the FBGs and infrared camera were consistent.

### 5.2. Measurement and Result Analysis of Spatial Temperature Field Near the Surface of Photovoltaic Module

In this experiment, FBGs were used to measure the spatial temperature field at point P14 on the surface of the photovoltaic module and its normal direction.

**Figure 8:**
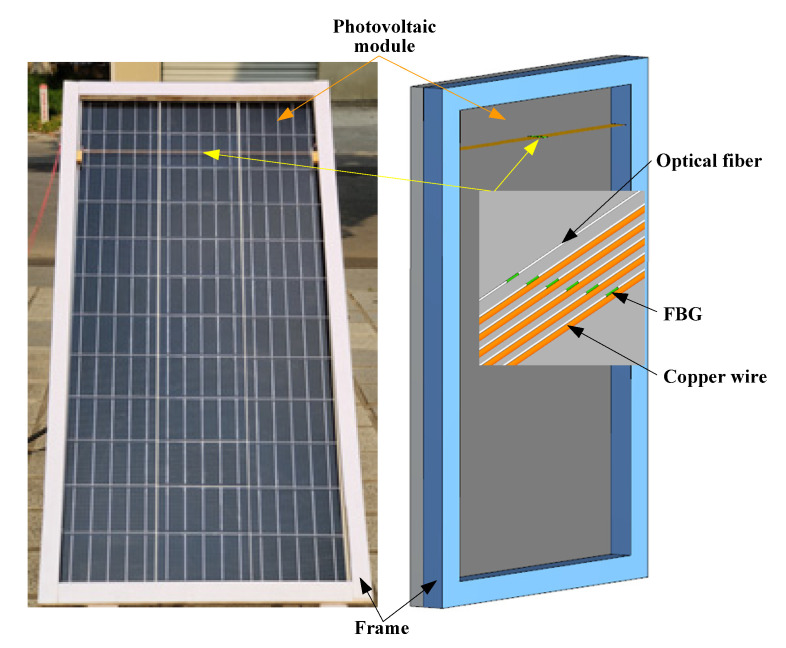
Schematic diagram of the spatial layout of FBG array.

The rectangular frame was close to the surface of the photovoltaic module, and the inner frame of the frame was the same size as the photovoltaic module. To prevent stress interference with the FBGs and maintain their normal spacing, a number of copper wires were fixed at the temperature measurement position inside the frame. The diameter of the copper wires was 0.15 mm, and they were arranged in parallel along the normal direction of the photovoltaic module, with a spacing of 2 mm. FBGs were applied on the surface of the photovoltaic module and copper wires with thermal conductive silicone grease. The spatial layout of the FBGs and measurement device is shown in Figure 8.

**Figure 9:**
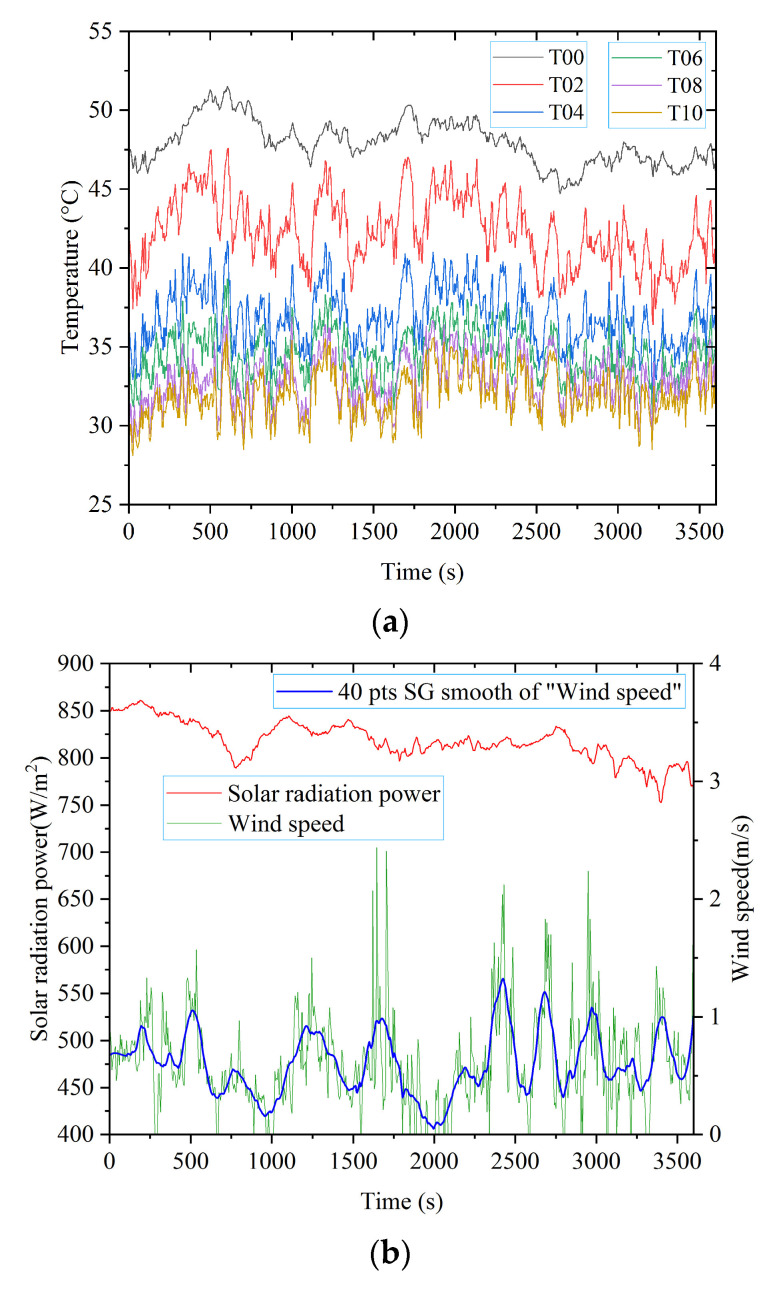
Results of spatial temperature field measurement experiment: (**a**) photovoltaic module temperature field; (**b**) solar radiation power and wind speed.

At 13:30 p.m., the photovoltaic module was placed outdoors in sunny weather with a tilt angle of 45°. The temperature data were measured by the FBG array. Then, they were recorded during the time period from 14:00 to 15:00, as shown in Figure 9. T00, T02, T04, T06, T08, and T10 were the temperatures at locations with a normal distance of 0, 2, 4, 6, 8, and 10 mm from point P14, respectively. T00 was the temperature at point P14 on the surface of the photovoltaic module. At the same time, the solar radiation power and wind speed of the experimental environment were recorded by an anemometer and solar power meter, respectively.

The measurement results were analyzed as follows:

(1) Analysis of the change law of the temperature field of the photovoltaic module

According to Figure 9, the temperature of the photovoltaic module decayed from the surface to space. The decay rate gradually decreased with distance.

The fluctuation law of the photovoltaic module surface and near-field spatial temperature was basically consistent with that of the solar radiation power.

Affected by wind speed, the near-field spatial temperature amplitude of the photovoltaic module widely varied with a high number of wave crests.

(2) Analysis of the influence of solar radiation power and wind speed on the temperature field of the photovoltaic module

**Figure 10:**
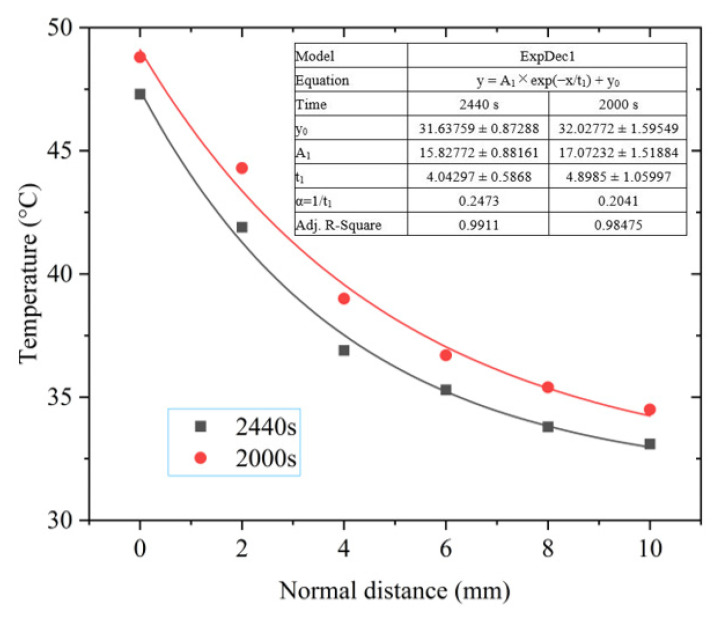
Temperature decay curve under different wind speeds.

The solar radiation power was 817 W/m^2^ at the time of 2000 s, and the solar radiation power was 820 W/m^2^ at the time of 2400 s, which are very close to each other. However, the wind speed at 2400 s was much higher than that at 2000 s. The spatial temperature distribution of the photovoltaic module at the two moments is shown in Figure 10.

To study the decay law of the spatial temperature, the first-order decay exponential function was used to fit the decay trend of the temperature, as shown in Figure 10. According to the fitting results, the adjusted fitting degree R^2^ was good, being greater than 0.98.

From Figure 9a and Figure 10, we observed that the temperature value sharply dropped within 6 mm from the photovoltaic module surface, and then the downward trend became flat. The temperature decay coefficient was α_1_ = 0.2041 at 2000 s and α_2_ = 0.2473 at 2440 s, α_1_ < α_2_. Therefore, the near-field spatial temperature decay rate of the photovoltaic module was very sensitive to wind speed, and the higher the wind speed, the faster the decay rate.

**Figure 11:**
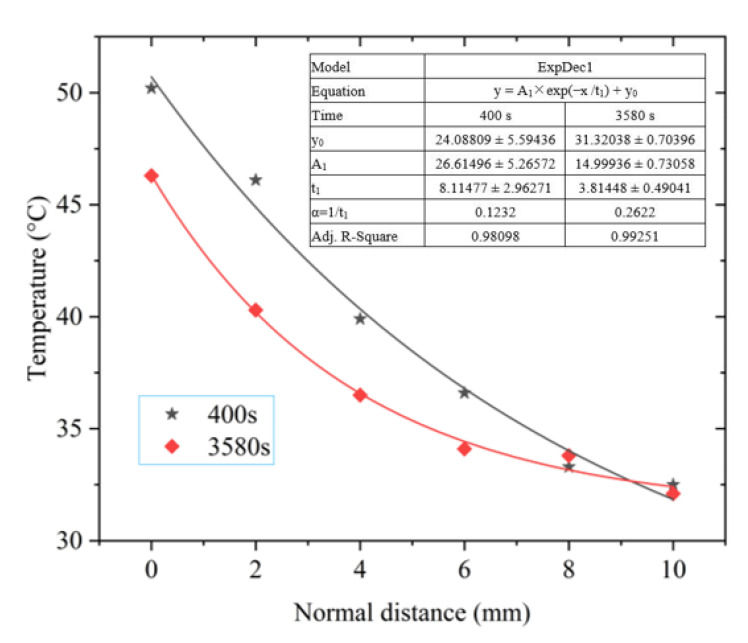
Temperature decay curve under different solar radiation power.

The wind speed values at 400 and 3580 s were small and close, but the solar radiation power at the two moments was quite different, 845 W/m^2^ at 400 s and 770 W/m^2^ at 3580 s, respectively. The spatial temperature distribution of the photovoltaic module at the two moments is shown in Figure 11.

From Figure 11 and the fitting results, we found that the greater the solar radiation power, the slower the downward trend in the near-field temperature of the photovoltaic module under the condition of close wind speed.

In the experiment, bare FBGs were used for temperature detection; therefore, the temperature sensitivity was higher, and the measured temperature value was more accurate. However, the bare optical fibers and FBGs were brittle and breakable, so care and caution should be taken during measurement operations. In engineering applications, the necessary encapsulation of FBGs is required.

## 6. Conclusions

In order to detect the surface and near-field spatial temperature of a photovoltaic module, we designed a temperature detection system based on multichannel FBG strings. Wavelength division multiplexing and space division multiplexing technologies were applied in this system. The FBG array was arranged on the surface and in the near-field of the photovoltaic module. The tunable laser method and peak-seeking algorithm were used to demodulate the wavelength displacement of each FBG. The temperature measurement and positioning of each point were realized.

An experimental platform was built to measure the temperature of a polycrystalline photovoltaic module in a sunny outdoor environment. The temperature distribution of the photovoltaic module surface was obtained. The temperature of the photovoltaic cell with hot spot failure was significantly higher than that of a normal working cell.

The fluctuation pattern of the photovoltaic module surface and near-field spatial temperature was basically consistent with the fluctuation pattern of the solar radiation power. The surface temperature of the photovoltaic module was less affected by wind speed than the near-field spatial temperature. The near-field spatial temperature of the photovoltaic module frequently fluctuated due to wind speed.

The temperature of the photovoltaic module decayed from the surface to space. Within 6 mm from the photovoltaic module surface, the temperature sharply dropped. Then, the downward trend became gentle. The photovoltaic module surface and near-field spatial temperature decay rate was very sensitive to wind speed; the higher the wind speed, the faster the decay. The closer the wind speed, the greater the solar radiation power, and the slower the temperature decay of the photovoltaic module.

## Figures and Tables

**Table 1:** Temperature data of each photovoltaic cell measured by FBGs.

Cell Number	Temperature (°C)	Cell Number	Temperature (°C)	Cell Number	Temperature (°C)
1	42.24	13	42.36	25	40.06
2	42.95	14	42.30	26	60.38
3	42.80	15	42.46	27	40.26
4	42.71	16	41.54	28	40.48
5	42.42	17	41.92	29	41.45
6	42.56	18	42.21	30	41.86
7	42.59	19	41.85	31	41.79
8	42.67	20	41.91	32	41.74
9	43.13	21	42.56	33	42.47
10	43.12	22	42.79	34	42.56
11	43.16	23	43.10	35	42.87
12	42.76	24	42.40	36	42.32
